# Maxillomandibular Advancement in the Management of Obstructive Sleep Apnea

**DOI:** 10.1155/2012/373025

**Published:** 2012-01-29

**Authors:** Ranji Varghese, Nathan G. Adams, Nancy L. Slocumb, Christopher F. Viozzi, Kannan Ramar, Eric J. Olson

**Affiliations:** ^1^Division of Pulmonary and Critical Care Medicine, Center for Sleep Medicine, Mayo Clinic, 200 1st Street SW, Rochester, MN 55905, USA; ^2^Division of Oral and Maxillofacial Surgery, Mayo Clinic, Rochester, MN 55905, USA

## Abstract

Maxillomandibular advancement (MMA) is a surgical option for obstructive sleep apnea (OSA). MMA involves forward-fixing the maxilla and mandible approximately 10 mm via Le Fort I maxillary and sagittal split mandibular osteotomies. We retrospectively reviewed outcomes from 24 consecutive OSA patients who underwent MMA at our institution. MMA resulted in an 83% reduction in the group mean apnea-hypopnea index (AHI) per polysomnography an average of 6.7 months after surgery. Forty-two percent of patients achieved a post-MMA AHI of less than 5 events/hour sleep and 71% achieved an AHI less than or equal to 10 events/hour sleep. The Epworth Sleepiness Scale score decreased by an average of 5 post-surgery. No parameters predictive of cure for OSA by MMA were identified.

## 1. Introduction

Obstructive sleep apnea (OSA) is a common disorder [1] associated with significant morbidity and mortality [2, 3]. Critical narrowing of the upper airway during sleep occurs behind the uvula and soft palate, at the base of the tongue, or at both sites and develops due to a dysfunctional interplay of anatomic factors narrowing the airway and compensatory neuromuscular mechanisms insufficient to maintain airway patency [4]. Continuous positive airway pressure (CPAP), which pneumatically splints the upper airway, is considered the therapeutic mainstay for OSA [5] yet a significant minority of patients struggle to adhere to this therapy [6]. Greater than 50% of patients experience CPAP-associated side effects that may be related to the prescribed pressure, interface, and/or equipment [7]. Accordingly, physicians may consider non-CPAP treatment alternatives for their OSA patients, including risk factor modifications [8], oral appliances [9], or a variety of surgical procedures performed singularly, sequentially, or simultaneously [10].

The role of upper airway surgery for OSA has been contentious [11, 12]. Most upper airway surgeries for OSA have limited success if stringent criteria are applied with respect to the apnea-hypopnea index (AHI), the traditional disease-defining statistic for OSA [13]. Uvulopalatopharyngoplasty (UPPP), perhaps the most common surgery performed for OSA, results in normalization of the AHI in less than 20% of patients [13]. Similarly, procedures such as nasal reconstruction, laser-assisted uvulopalatoplasty, hyoid suspension, and radiofrequency ablation of upper airway structures are generally not curative options for OSA [10].

A surgical procedure with an apparently greater efficacy for lowering the AHI is maxillomandibular advancement (MMA) which enlarges the upper airway and decreases upper airway collapsibility [14] by forward-fixing the maxilla and mandible approximately 10 mm via Le Fort I maxillary and sagittal split mandibular osteotomies [15]. The potential advantages of MMA include a single-stage surgery to address retropalatal and retrolingual obstruction and preservation of pharyngeal tissue functional integrity [16]. Though edema and hypopharyngeal hematoma may occur in the immediate postoperative period following MMA, there appears to be a minimal risk for airway compromise or postoperative exacerbation of OSA [17]. Indeed, CPAP-like reductions in the AHI of nearly 90% with MMA have been reported [16, 18–25] which have been associated with improvements in sleep architecture [16, 18, 21, 23] and daytime vigilance [23], though these studies had some methodologic limitations [13].

In an effort to further define the role for MMA in OSA, we sought to review our experience with MMA. In doing so, we also wanted to address some of the limitations in the previously performed studies, such as controlling for body mass index (BMI) pre- and post-MMA and the percent of time spent supine in the pre- and post-MMA polysomnograms, examining changes in the pre- and post-MMA subjective sleepiness, and presenting tiered AHI outcomes. In addition, we sought to identify pre-surgical parameters predictive of surgical success.

## 2. Methods

After obtaining approval from the Mayo Clinic Institutional Review Board, we retrospectively reviewed records of consecutive patients, 18 years or older, who underwent MMA for the indication of OSA and had pre- and post-MMA polysomnograms (PSGs) between November 1, 2004 and June 30, 2008. Patients with persistent OSA despite previous surgical interventions as well as surgery naïve patients were included. Pre- and post-MMA clinical, demographic, and PSG data were collected by review of each patient's comprehensive electronic medical record.

### 2.1. Patient Selection

Patients reported herein were accrued from routine clinical practice and typically had moderate to severe OSA for which they could not or would not use CPAP. All underwent a multidisciplinary preoperative evaluation, including cephalometric radiographs and examinations by an oral and maxillofacial surgeon (CFV) and an ear, nose, and throat (ENT) specialist (20 patients; the remaining 4 had either been seen by an ENT specialist elsewhere and were not deemed a candidate for nasal and/or palatal surgery or had already undergone nasal and/or palatal surgery), and a sleep specialist. The ENT exam included flexible fiberoptic nasal endoscopy and laryngoscopy. On the basis of clinicoradiologic data, patients who eventually underwent MMA were not felt to have significant nasal or palatal obstruction and were free of significant co-morbidities that might negatively impact surgical risk, such as unstable cardiopulmonary disease.

### 2.2. Polysomnography

All patients were evaluated preoperatively by a board-certified/board eligible sleep physician at our American Academy of Sleep Medicine (AASM)-accredited sleep facility. All PSG studies were technologist-attended, in-laboratory examinations using a digital polygraph. For the PSGs performed at our facility, the following parameters were recorded: electroencephalography (F_z_-C_z_, C_z_-O_z_, C_4_-M_1_, or C_3_-M_2_); electrooculography (right outer canthus-F_pz_; left outer canthus-F_pz_); submental and anterior tibialis electromyography; snoring by laryngeal microphone; electrocardiography; pulse oximetry; respiratory effort (thoracic, abdominal, and summated inductive plethysmography). Until August 2006, airflow was analyzed by nasal pressure transducer. From September 2006 onward, airflow was analyzed by both oronasal thermal sensor and nasal pressure transducer. Obstructive apnea was defined as cessation of airflow for at least 10 seconds despite respiratory effort. Hypopnea was defined by a ≥30% reduction in airflow for at least 10 seconds accompanied by at least a 4% drop in SpO_2_. Until July 2007, arousals were scored according to American Sleep Disorders Association criteria [26] and sleep stages per Rechtschaffen and Kales [27]. Thereafter, arousals and sleep stages were scored per the AASM scoring manual [28].

Data on the following pre- and post-MMA PSGs parameters (when available) were collected: AHI (number of apneas and hypopneas/hour of sleep); sleep efficiency (total sleep time/time in bed); percentages of nonrapid eye movement (NREM) and rapid eye movement (REM) sleep; percentage of time spent in the supine position; initial sleep onset latency; initial REM latency; lowest oxyhemoglobin saturation (LSAT); mean oxyhemoglobin saturation; arousal index (number of arousals/hour of sleep); respiratory-related arousal index (number of respiratory-related arousals/hour of sleep). Arousals were deemed respiratory-related if immediately preceded by apneas, hypopneas, periods of at least 10 seconds of diminished airflow that did not meet criteria for hypopnea. Patient sleep position was assessed by the attending technologist via continuous video monitoring.

### 2.3. Subjective Sleepiness

Subjective sleepiness pre- and post-MMA was assessed via the Epworth Sleepiness Scale (ESS) [29]. Using this questionnaire, patients rate their ease of falling asleep in 8 potentially sleep-permissive situations using a 0 (no chance of dozing) to 3 (high chance of dozing) ranking. The numbers for each scenario are added to obtain a total score. Scores of 0–9 are considered normal while scores of 10–24 imply excessive subjective sleepiness.

### 2.4. Cephalometric Radiographs and Analysis

**Figure 1:**
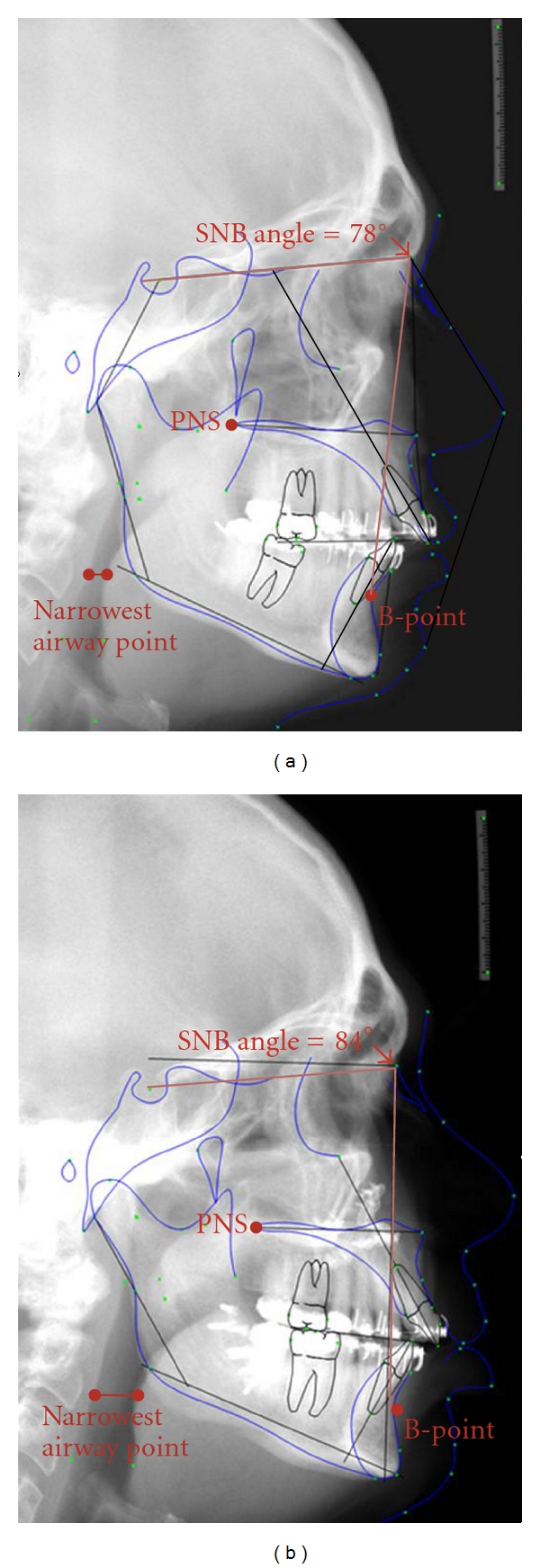
Pre- (a) and post- (b) maxillomandibular advancement (MMA) lateral cephalometric radiographs demonstrating analyzed parameters. Pre- and post-MMA cephalometric images were digitally overlaid to determine the change in position of the following parameters: anatomic B-point (most posterior midline point in the anterior concavity of the mandible); posterior nasal spine (PNS) along the sella-nasion line; and sella-nasion-point B (SNB) angle. Enlargement of the posterior airway space was also determined on all cephalometric radiographs by measuring the narrowest point in the airway pre- and post-MMA, then subtracting the larger (post-MMA) value from the smaller (pre-MMA) value to obtain the difference.

Lateral cephalometric radiographs were obtained on each patient preoperatively and within 6 months postoperatively. All radiographs were taken by the same technician with patients in the natural resting head position, not swallowing, and in complete intercuspation. The phase of respiration was not controlled during attainment of the lateral cephalometric radiographs. Pretreatment and posttreatment radiographs were taken on the same cephalostat by the same operator. The digital lateral cephalograms were transferred to Dolphin imaging and analysis software (Patterson Technology, Chatsworth, CA). All radiographs were traced by the same surgeon to ensure proper cephalometric point selection and eliminate measurement acquisition errors. Linear magnification of the cephalostat was 10%. Pre- and post-MMA cephalometrics were digitally superimposed using best fit of unchanged skull base anatomy to align images in order to determine millimetric changes in postoperative position of the following points (Figure 1): anatomic B-point (most posterior midline point in the anterior concavity of the mandible) to determine mandibular movement and change in position of the genioglossus muscle; posterior nasal spine (PNS) along the sella-nasion line to assess maxillary movement; sella-nasion-point B (SNB) angle to evaluate change in mandibular position. Enlargement of the posterior airway was also determined on all cephalometric radiographs by manual measurement of the narrowest point in the airway pre- and post-MMA (Figure 1).

### 2.5. Maxillomandibular Advancement Description

Prior to the surgical procedures, all patients had intraoperative surgical guide splints constructed from occlusal models to allow an accurate advancement of 10 mm for the maxilla and mandible. All surgeries were performed at our institution by a single surgeon (CFV) under general anesthesia in the operating room. Hypotensive anesthesia was utilized to minimize blood loss. The maxillary osteotomy was performed first and was at the LeFort I level. Minimal mucosal stripping and preservation of blood vessels was performed to minimize risk of devascularization of osseous segments. The maxilla was advanced 10 mm and secured in place with titanium miniplates and multiple screws. Once this was done, bilateral mandibular osteotomies were performed using a sagittal splitting technique to allow the mandible to be advanced. The mandible was then secured with multiple bicortical position screws. After confirmation of proper bone position and occlusal accuracy, the wounds were closed. Patients were not wired shut; mobility and immediate function (soft diet limitation) were allowed.

Our patients were all routinely placed in the ICU overnight to monitor for airway embarrassment due to swelling. The next morning, they were transferred to a regular room and allowed to begin oral feeds and oral pain management, as well as ambulation. None of our patients demonstrated airway compromise in the perioperative period, nor did any require reintubation or CPAP/bilevel PAP. Soft diet was continued for six weeks after discharge from the hospital. Close followup was completed in all patients (one week, three weeks, and six weeks after discharge).

### 2.6. Statistical Analysis

Data were analyzed using JMP software (SAS Institute, Cary, NC, USA). Comparisons of continuous variables were performed using a two-tailed *t*-test or Wilcoxon rank-sum test as appropriate, and categorical responses were compared using chi-square analysis. Paired-sample *t* tests were used to compare pre- and postsurgery data in Table 1. Only data that were available both before and after MMA were used in the paired analysis. *P* < 0.05 was considered statistically significant. Data are summarized as mean ± standard deviation.

## 3. Results

The records of twenty-four OSA patients (18 men [75%]) treated with MMA and with pre- and postoperative PSG data were identified and analyzed. The mean age at the time of surgery was 48.3 ± 10.8 years. All patients were Caucasians.

Group pre- and post-MMA PSG data are provided in Table 1. Twelve patients had their pre-MMA PSG elsewhere and their post-MMA at our facility, while 12 patients underwent both pre- and post-MMA PSG at our facility. PSGs were performed 25.9 ± 29.1 months before and 6.7 ± 3.8 months after MMA. The mean absolute decrease in the AHI after MMA was 37.6 ± 5.9 events/hour sleep (CI −25.2 to −49.9) (*P* < 0.001) for an average AHI reduction of 83%. Ten (42%) patients achieved an AHI < 5 events/hour sleep; 17 (71%) patients achieved an AHI ≤ 10 events/hour sleep; 21 (87.5%) patients achieved a 50% AHI reduction and/or an AHI ≤ 20 events/hour sleep. The mean ESS score decreased by 35% after MMA. The percentages of stages N3 and REM sleep, the LSAT, and the respiratory-related arousal index improved during post-MMA PSG. Neither post-MMA BMI nor the percentage of supine position sleep during the post-MMA PSG was significantly different from pre-MMA values.

Table 2 provides patient-specific data on pre-MMA upper airway surgeries, maxillary and mandibular advancement, and changes in AHI. Eight (30%) patients had previous upper airway surgeries.

All but one patient enjoyed an AHI reduction. This patient was a 61-year-old man with OSA and multifactorial insomnia. His BMI was normal (24 kg/m^2^), and his oropharynx configuration was Grade II per modified Mallampati technique [30]. The preoperative AHI was 26 events/hour sleep; the postoperative AHI was 42 events/hour sleep. The outcome could not be explained by differences in weight, the frequency of central apneas, or the amount of supine position sleep during PSG. He eventually underwent hyoid suspension at another institution with a resultant drop in his AHI to 15 events/hour sleep. This patient had a presurgical Class III malocclusion (underbite) and had been treated with orthodontics elsewhere prior to presenting to us for surgery. Surgical correction of the class III malocclusion resulted in less anterior movement of the mandible than is typical in MMA surgery. The mandibular advancement was only 3 millimeters. This lesser movement most likely was the cause of the poor response to MMA in this isolated patient.

Comparing various parameters between those cured of OSA by MMA (defined by postoperative AHI < 5 events/hour sleep) versus those with residual sleep disordered breathing, no significant differences were found with respect to age, gender, pre- or postoperative BMI, prior upper airway surgery, amount of maxillomandibular advancement, or cephalometric measurements (Table 3).

## 4. Discussion

This study adds to the literature that MMA results in a substantial reduction of the AHI in the majority of nonrandomized patients described in case series. In our study population of patients with generally severe OSA, there was an 83% drop in the mean AHI, a value congruent with the 87% reduction by MMA per a recent review of upper airway surgeries for OSA sponsored by the AASM [31] and the 85% reduction reported in an MMA meta-analysis by Holty and Guilleminault [32]. The latter publication also reported an average residual AHI of 9.5 ± 10.7 events/hour sleep, which compares to an average residual AHI of 7.8 events ± 10.5 events/hour sleep in our group with equally severe OSA.

There has been a call [13] for use of more stringent criteria (i.e., postsurgical AHI < 5 events/hour sleep) for judging success of surgical treatment for OSA beyond the traditional definition of at least a 50% reduction in AHI and residual AHI < 20 events/hour sleep. With this in mind, we herein report our AHI outcomes in a tiered manner with 10 (42%) patients achieving a post-MMA AHI < 5 events/hour sleep and 17 (71%) patients reaching a post-MMA AHI < 10 events/hour sleep. Similarly, Holty and Guilleminault [32] reported that 43% and 63% of patients in their meta-analysis had AHI < 5 and AHI < 10, respectively, after MMA.

The MMA meta-analysis [32] found that younger patient age, lower preoperative weight, lower preoperative AHI, and greater degree of maxillary advancement were predictive of increased MMA success. We did not find any variables that were predictive of success in our group. There were no significant differences in age, gender, BMI, preoperative AHI, amount of advancement of the jaws, or cephalometric parameters. We suspect that our inability to detect critical clinical, surgical, and cephalometric thresholds for success was due to our limited population size.

Determining the role for any upper airway surgery in the management of OSA has been complicated by methodologic limitations in the surgical literature, including irregularities in the performance and interpretation of PSG (such as controlling for the percent sleep time in the more vulnerable supine position), lack of secondary outcome data beyond AHI (such as sleep architecture and ESS), and failure to control for the potential confounding of peri-procedural changes in BMI [12, 13]. This study attempted to address these issues when possible. The percentage of supine position sleep was controlled for when comparing the pre- and post-MMA AHI results, the first such time that we know of in the MMA literature. As there were no significant differences, our MMA results were not positively influenced by decreased supine sleep on the postoperative PSG. Pertaining to outcomes of MMA beyond impact on the AHI, our report adds to the much smaller body evidence on post-MMA sleep architecture and subjective sleepiness (sleep architecture data were available in only 46% of patients in the Holty and Guilleminault's meta-analysis [32] of 627 patients and ESS data in just 7%). Our sleep architecture improvements mirror those reported by others [16, 18, 21, 23], while the pre- and post-MMA ESS improvement data are also similar. The group mean ESS score drop by 5 in our series is a reduction at least as strong as reported with CPAP in patients with severe OSA [33]. BMI was accounted for pre- and post-MMA and did not change significantly suggesting that success of MMA could not simply be attributed to weight loss after surgery.

This study shares a number of limitations common in the OSA surgical literature. Our patients were not randomized to MMA, although there are significant practical hurdles to randomization in surgical trials for OSA, especially with an invasive surgery with cosmetic ramifications such as MMA. Not all patients who underwent MMA at our institution during the data collection period underwent postprocedural PSG. We did not have pre- and post-MMA matched data for every parameter for every patient either because the pre-MMA PSG was performed elsewhere or the documentation was incomplete, a problem inherent in retrospective analyses. In the case of patients who had their pre-MMA PSG at another institution, a repeat pre-operative study was not performed because it was either deemed clinically unnecessary or logistically impractical. Systematic documentation of post-MMA complications was not performed so adverse outcomes are not reported herein.

Our study population included 8 patients who had previously undergone surgeries for OSA, mostly UPPP, which limits our ability to discern the specific impact of MMA. The patient numbers were too small to determine the impact of the specific previous upper airway surgeries on response to MMA. However, the frequency of previous surgery likely reflects, at least in part, the enduring paradigm of a step-wise surgical approach to OSA in which site-specific phase I surgery addresses areas deemed susceptible to obstruction in the naso-, oro-, or hypopharyngeal regions, while phase II surgery consists of facial skeletal advancement (i.e., MMA) for residual disease [34]. Debate continues about how best to determine a successful outcome from OSA treatment. Using the admittedly narrow but important endpoint of impact on AHI, the pooled success rate for phase I surgeries achieving an AHI ≤ 10 events/hour sleep is 31%, and an AHI of ≤5 events/hour sleep is 13% [13]. Considering that the impact of MMA on AHI is more substantial, the results have been replicated at multiple institutions and that the AHI response appears durable for at least at 2 years [18], an argument can be made for proceeding directly to MMA in patients seeking a surgical approach to their OSA.

## 5. Conclusion

CPAP remains the standard treatment for most patients with OSA [5]. However, MMA provides an alternative for OSA patients who cannot or will not use CPAP. Experience is growing with this procedure which yields a significant drop in AHI and is associated with improvements in sleep architecture and subjective sleepiness.

## Figures and Tables

**Table 1:** Group pre- and post-maxillomandibular advancement (MMA) demographic- and polysomnographic-matched pair data.

	*n*	Pre-MMA (mean ± SD)	Post MMA (mean ± SD)	*P* value
ESS	14	13.6 ± 5.4	8.8 ± 3.3	0.0063**
BMI (kg/m^2^)	18	30.5 ± 6.0	30.3 ± 5.3	0.7
AHI (number/hour)	24	45.4 ± 26.4	7.8 ± 10.5	<0.0001**
Sleep efficiency (%)	17	72.2 ± 18.9	78.6 ± 16.7	0.2
Stage N1 (%)	15	22.3 ± 15.9	14.2 ± 11.2	0.08
Stage N2 (%)	15	55.4 ± 12.1	55.3 ± 8.3	0.97
Stage N3 (%)	15	10.7 ± 10.2	15.3 ± 8.3	0.04**
Stage R (%)	16	9.1 ± 7.2	15.2 ± 6.9	0.0078**
Respiratory-related arousals (events/hour)	12	49.0 ± 21.4	11.5 ± 14.9	0.0002**
Lowest oxyhemoglobin saturation (%)	21	81.2 ± 8.0	86.2 ± 5.6	0.03**
Time in supine position (%)	12	44.4 ± 28.5	41.7 ± 28.9	0.7

Abbreviations: SD: standard deviation; ESS: Epworth Sleepiness Scale; BMI: body mass index (kg/m^2^); AHI: apnea-hypopnea index (apneas and hypopneas per hour of sleep); N: non-rapid eye movement sleep; R: rapid eye movement sleep.

**Table 2:** Selected, patient-specific data on demographics, previous airway surgery, surgical advancement, and sleep-disordered breathing pre- and post-maxillomandibular advancement (MMA).

Patient	Gender	Pre-MMA upper airway surgery	Age at MMA	Maxillary advancement (mm)	Mandibular advancement (mm)	Pre-MMA AHI	Post-MMA AHI	% AHI reduction
1	F	None	39	8	7	24	1	96
2	M	None	45	NA	NA	30	1	97
3	F	None	57	9	9	21	11	48
4	M	UPPP, bilateral tonsillectomy	48	8	8	37	11	70
5	M	None	61	10	3	26	42	—
6	M	UPPP	36	10	10	80	1	99
7	M	None	35	6	6	23	1	96
8	M	UPPP, Genioglossus advancement	33	10	10	38	0	100
9	F	None	55	10	10	80	11	86
10	M	UPPP, nasal septoplasty	54	10	10	37	1	97
11	M	None	55	7	7	8	1	88
12	M	None	42	10	10	12	5	58
13	M	None	57	8	8	54	5	91
14	M	None	49	10	10	52	2	96
15	F	UPPP, nasal septoplasty	48	10	10	56	1	98
16	M	None	56	10	10	37	9	76
17	M	None	45	10	10	25	5	80
18	M	UPPP	44	12	12	117	1	99
19	M	None	26	NA	NA	33	11	67
20	M	UPPP, nasal septoplasty	50	10	10	39	7	82
21	F	None	71	NA	NA	53	14	74
22	M	None	30	10	10	95	6	94
23	F	None	58	10	10	51	36	29
24	M	Genial advancement	51	10	10	60	5	92

Abbreviations: F: female; M: male; UPPP: uvulopalatopharyngoplasty; AHI: apnea-hypopnea index (apneas and hypopneas/hour sleep); NA: data not available.

**Table 3:** Comparison of parameters between patients cured versus those with residual obstructive sleep apnea following maxillomandibular advancement (MMA).

	Post-MMA				
	Surgical cure (AHI < 5) *N* = 10; 42% (mean ± SD)	*n*	No cure (AHI ≥ 5) *N* = 14; 58% (mean ± SD)	*n*	*P* value
Pre-MMA				14	
Age (years)	44.4 ± 7.8	10	51.0 ± 12.0	14	0.07
Male (%)	80	8	71.4	10	0.5
BMI	30.3 ± 4.4	9	31.0 ± 6.5	12	0.9
AHI (events/h)	46.5 ± 31.9	10	44.6 ± 23.0	14	0.9
SpO_2_ nadir (%)	81.1 ± 9.6	10	81.3 ± 6.6	12	0.7
Previous phase-I surgery^1^ (%)	62.5	5/8	37.5	3/8	0.2

Surgery					
Maxillary advancement (mm)	9.2 ± 1.9	9	9.6 ± 0.8	12	0.8
Mandibular advancement (mm)	9.1 ± 2.0	9	9.0 ± 2.0	12	0.9

Post-MMA					
BMI (kg/m^2^)	29.2 ± 7.1	8	30.0 ± 3.8	13	0.9
% Change in BMI (%)	−1.7 ± 5.5	7	0.5 ± 6.7	11	0.6
AHI (events/h)	1.0 ± 0.5	10	12.7 ± 11.6	14	<0.0001
% Change in AHI (%)	−96.5 ± 3.5	10	−63.2 ± 40.1	14	<0.0001
SpO_2_ nadir (%)	88.8 ± 4.2	10	84.1 ± 5.2	14	0.0221
% Change in SpO_2_ nadir (%)	10.9 ± 14.9	10	3.9 ± 12.8	12	0.2

Abbreviations: SD: standard deviation; BMI: body mass index (kg/m^2^); AHI: apnea-hypopnea index (apneas and hypopneas/hour sleep); SpO_2_: oxyhemoglobin saturation.

^1^Patients who underwent an upper airway procedure prior to MMA.
